# Unveiling Shared Immune Responses in Porcine Alveolar Macrophages during ASFV and PRRSV Infection Using Single-Cell RNA-seq

**DOI:** 10.3390/microorganisms12030563

**Published:** 2024-03-12

**Authors:** Bo Jiang, Lu Li, Yu Wu, Xiaoying Wang, Ning Gao, Zhichao Xu, Chunhe Guo, Sheng He, Guihong Zhang, Yaosheng Chen, Xiaohong Liu, Zhengcao Li

**Affiliations:** 1 State Key Laboratory of Biocontrol, School of Life Sciences, Sun Yat-Sen University, Guangzhou 510006, China; jiangb27@mail3.sysu.edu.cn (B.J.); lilu75@mail2.sysu.edu.cn (L.L.); wuyu65@mail2.sysu.edu.cn (Y.W.); xiaoyingwang11@163.com (X.W.); gaon@hunau.edu.cn (N.G.); xuzhich5@mail.sysu.edu.cn (Z.X.); guochunh@mail.sysu.edu.cn (C.G.); hesh56@mail3.sysu.edu.cn (S.H.); chyaosh@mail.sysu.edu.cn (Y.C.); xhliu@163.net (X.L.); 2 Research Center for African Swine Fever Prevention and Control, South China Agricultural University, Guangzhou 510642, China; guihongzh@scau.edu.cn; 3 School of Biology, Jiaying University, Meizhou 514015, China

**Keywords:** pigs, single-cell RNA sequencing (scRNA-seq), ASFV, PRRSV, PAMs, immune responses

## Abstract

African swine fever virus (ASFV) and porcine reproductive and respiratory syndrome virus (PRRSV) infections lead to severe respiratory diseases in pigs, resulting in significant economic losses for the global swine industry. While numerous studies have focused on specific gene functions or pathway activities during infection, an investigation of shared immune responses in porcine alveolar macrophages (PAMs) after ASFV and PRRSV infections was lacking. In this study, we conducted a comparison using two single-cell transcriptomic datasets generated from PAMs under ASFV and PRRSV infection. Pattern recognition receptors (PRRs) RIG-I (DDX58), MDA5 (IFIH1), and LGP2 (DHX58) were identified as particularly recognizing ASFV and PRRSV, triggering cellular defense responses, including the upregulation of four cytokine families (CCL, CXCL, IL, and TNF) and the induction of pyroptosis. Through weighted gene co-expression network analysis and protein–protein interaction analysis, we identified thirteen gene and protein interactions shared by both scRNA-seq analyses, suggesting the ability to inhibit both ASFV and PRRSV viral replication. We discovered six proteins (PARP12, PARP14, HERC5, DDX60, RSAD2, and MNDA) in PAMs as inhibitors of ASFV and PRRSV replication. Collectively, our findings showed detailed characterizations of the immune responses in PAMs during ASFV and PRRSV infections, which may facilitate the treatments of these viral diseases.

## 1. Introduction

African swine fever (ASF) and porcine reproductive and respiratory syndrome (PRRS) are two of the most widespread viral diseases, inflicting substantial economic losses upon the global swine industry [1,2,3]. ASF virus (ASFV) is characterized as a double-stranded DNA virus belonging to the *Asfarviridae* family [4], while PRRS virus (PRRSV) is identified as a single-stranded positive-sense RNA virus belonging to the *Arteriviridae* family [5]. Although there are differences in the genetic structure of these two viruses, their mature virions share the common feature of being enveloped with capsid proteins, marking the completion of viral assembly. Specifically, p72 serves as the capsid protein in ASFV, while N proteins fulfill this role in PRRSV [6,7]. Both viruses have a highly restricted cell tropism for porcine alveolar macrophages (PAMs) [8,9,10]. Under non-infected conditions, macrophages maintain the M0 type [11]. However, upon alteration of the microenvironmental signals in response to virus infection, the immune responses of macrophages are triggered. Cells undergoing viral infection express germline-encoded pattern recognition receptors (PRRs) to recognize unique microbial components, known as pathogen-associated molecular patterns (PAMPs) [12,13]. Upon recognition, PRRs activated intracellular signaling pathways leading to a series of immune responses, including elevated cytokines and the secretion of interferon (IFN) [14]. Numerous reports have detailed the immune responses of PAMs exposed to the two viruses. ASFV infection is associated with the regulation of chemokine expression and the upregulation of various cytokine families [15]. Conversely, PRRSV infection can activate PRRs and produce IFNs to inhibit virus replication [16]. However, these studies primarily focused on specific genes or pathways, creating a gap in the understanding of the common immune response in PAMs infected with both viruses. Thus, comprehensive research on the immune responses of PAMs is deemed crucial for understanding ASFV and PRRSV pathology.

Single-cell sequencing technologies have proven to be invaluable assets in virology, enabling the investigation of responses to viral infections at the single-cell level [17,18]. Utilizing single-cell transcriptome technology, macrophages have been characterized for their diverse immune responses in various viral infections, including HIV [19], influenza virus [20], COVID-19 [21], and more. Weighted gene co-expression network analysis (WGCNA) enables the examination of gene co-expression and the revelation of interactions between genes based on their expression levels [22]. The feasibility of applying WGCNA analysis at the single-cell level has been demonstrated, leveraging the wealth of information available from the samples [23]. To date, scRNA-seq research has been conducted on bronchoalveolar lavage fluid (BALF) cells in ASFV infection, providing insights into the host responses of porcine alveolar macrophages (PAMs) and complex virus–host interactions [24]. However, there have been no studies investigating the immune responses of PAMs at the single-cell level following PRRSV infection.

In this study, we sequenced BALF cells infected by a highly pathogenic strain of PRRSV(HP-PRRSV) in vitro and downloaded the scRNA-seq dataset for ASFV infection. We then characterized the common immune responses of PAMs caused by the infection of ASFV and PRRSV by WGCNA analysis and protein–protein interaction analysis at the single-cell level. Our results reinforced the detailed characterizations of the viral infection process and discovered certain inhibitions of viral replication in the host cells. Our findings contributed to a unique understanding of the macrophage immunoreaction caused by ASFV and PRRSV infection, shedding light on the pathological mechanisms and facilitating the development of effective vaccines and antiviral drugs.

## 2. Materials and Methods

### 2.1. Single-Cell RNA Sequencing Datasets

In this study, we had access to two distinct sources of single-cell transcriptome datasets pertaining to viruses infecting porcine alveolar macrophages (PAMs). The ASFV dataset was obtained from a previously published study, while the PRRSV dataset was generated through experiments as part of our research. The single-cell transcriptome dataset for ASFV (GSE168113) was downloaded from NCBI at https://www.ncbi.nlm.nih.gov/geo/query/acc.cgi?acc=GSE168113 and was accessed on 5 May 2022, providing the raw expression matrix for subsequent analyses [24]. Concerning PRRSV, the elaborate experimental procedures were described in detail below. The specifics of both datasets are outlined in Table 1.

### 2.2. Harvesting BAL Fluid Cells and Challenge with PRRSV in Cells

Bronchoalveolar lavage fluid (BALF) cells were harvested from a healthy piglet devoid of viral infection, and the lungs were removed in a sterile environment. Sterile phosphate buffer solution (PBS) (Corning, New York, NY, USA) was injected through the tracheal route and subsequently collected. BALF cells were cultured in RPMI 1640 medium (Gibco, Waltham, MA, USA), supplemented with 10% (*v*/*v*) heat-inactivated fetal bovine serum (FBS) (Gibco, USA), and 100 U/mL penicillin and 100 μg/mL streptomycin (Gibco, USA). Purified BALF cells were pre-plated in a 35 mm culture dish. Then, the cells were infected with PRRSV at a multiplicity of infection (MOI) of 1, utilizing the JXA1 viral strain for in vitro infection experiments [25]. Four designated time points (6 h, 12 h, 24 h, and 36 h) were established for infection. The number of cells collected per experimental group was no less than 15,000, including the uninfected control sample. To ensure a cell viability of over 90%, the collected cells were preserved at −80 for subsequent single-cell sequencing.

### 2.3. Library Preparation, Sequencing, and Reads Mapping

The Chromium Single Cell 3′ v.3 assay (10× Genomics) was employed for the generation of sequencing libraries. Libraries were sequenced by the NovaSeq 6000 S2 platform (Illumina, San Diego, CA, USA) to achieve a depth of approximately 300 million reads per library with a 2 × 150 read length. All raw sequence reads were processed using CellRanger version 3.1.0. The Sscrofa 11.1 genome with annotation v11.1.104 and the PRRSV JXA1 genome were integrated together using cellranger mkref. The raw expression matrix was generated with the default parameters in commands cellranger count and cellranger aggr.

### 2.4. Quality Control, Cell Clustering, and Annotation

The raw expression matrices for both ASFV and PRRSV datasets were imported into R using the R package Seurat (v.4.0.1) and subjected to quality control (QC) procedures [26]. For the ASFV dataset, QC standards involved removing cells with <500 transcripts detected, >7500 transcripts detected, and >10% mitochondrial gene, accounting for differences in sequencing quality. For the PRRSV dataset, the standards were set to remove cells with <500 transcripts detected, >3000 transcripts detected, and >10% mitochondrial gene. The IntegrateData function was employed to integrate the expression matrices for both datasets with k.filter equal to 30. Calculations of variable genes of the top 2000 and 20 principal components were used for cell clustering. The crucial parameter resolution in BALF cell clustering for both datasets was set to a resolution of 0.2, while in PAMs clustering, resolution was 0.4. The visualization of cell clustering was performed with t-Distributed Stochastic Neighbor Embedding (t-SNE) dimensional reduction. Cluster markers were calculated by the FindAllMarkers function, and cell types were manually annotated based on the cluster markers with the top 10 log-fold change and a Bonferroni-adjusted *p*-value of <0.05.

### 2.5. Weighted Gene Co-Expression Network Analysis (WGCNA)

Cells expressing the viral P72 protein in the ASFV dataset and those expressing the viral N protein in the PRRSV dataset were merged using the IntegrateData function. Subsequently, gene co-expression networks were constructed using the R package WGCNA (v.1.72.1) on the expression matrix of these cells [22]. The detailed steps are as follows: (1) pre-processing for cells and genes: an equal proportion of cell compression was carried out according to cell clustering (resolution = 0.1), with the compression proportion set to 30 times. The mean value of genes in cells before compression was calculated as the gene expression value in cells after compression. Calculation of high variable genes of the top 3000 and screening of genes with the top 75% of the absolute deviation of the median. (2) Excluding outline samples and identifying the optimal soft threshold value: the function hclust was used to calculate the distance between samples, filtering out abnormal samples. The function pickSoftThreshold was employed to compute the R2 value, representing the fitting degree of the scale-free network. The power at which R2 reached 0.85 for the first time was considered the best soft threshold. (3) Obtaining the gene co-expression modules and calculating module correlations: the function blockwiseModules was utilized to obtain gene co-expression modules, with parameters minClusterSize set to 10 and deepSplit set to 2. Calculation of correlation coefficients between gene modules and drawing a heatmap of gene correlation was performed using the R package ggplot2 (v3.3.3) [27]. (4) Calculating the correlation between modules and cellular traits: Pearson correlation coefficients between the modules and cellular traits were computed, and the correlation was considered significant at *p* < 0.05. Cellular traits contained the Seurat cluster, mitochondrial gene expression, viral gene expression, and others.

### 2.6. KEGG Pathway Enrichment Analyses and Protein–Protein Interaction (PPI) Analysis

Cluster-specific expression markers were subjected to KEGG pathway enrichment analysis, employing Fisher’s exact test on curated gene sets [28]. Representative terms were chosen from the top 25 *p*-values to draw enrichment bar plots using the R package ggplot2. For PPI analysis, the STRING web tool (https://cn.string-db.org/) was utilized and was accessed on 16 December 2023. To obtain the local network cluster, the PPI network was clustered according to biological processes in Gene Ontology (GO) terms [28,29].

### 2.7. Quantitative Reverse Transcription–Polymerase Chain Reaction (qRT-PCR)

Total RNA was extracted from PAMs using TRIzol reagent (Invitrogen, Waltham, MA, USA), and then, cDNA was generated from total RNA with a Reverse Transcription System (Magen, Netanya, IL, USA) for real-time quantitative reverse transcriptase PCR (qRT-PCR) analysis, according to the manufacturer’s instructions. To quantify the load of the interested genes, relative qRT-PCR was performed using a Light-Cycler 480 PCR system (Roche, Basel, Switzerland). The expression of target genes was normalized to the endogenous reference gene GAPDH, using the threshold cycle (2^−ΔΔCt^) method. The qRT-PCR primers used above are shown in Table 2.

## 3. Results

### 3.1. Single-Cell Transcriptome Landscape in ASFV-Infected and PRRSV-Infected BALF Cells

**Figure 1:**
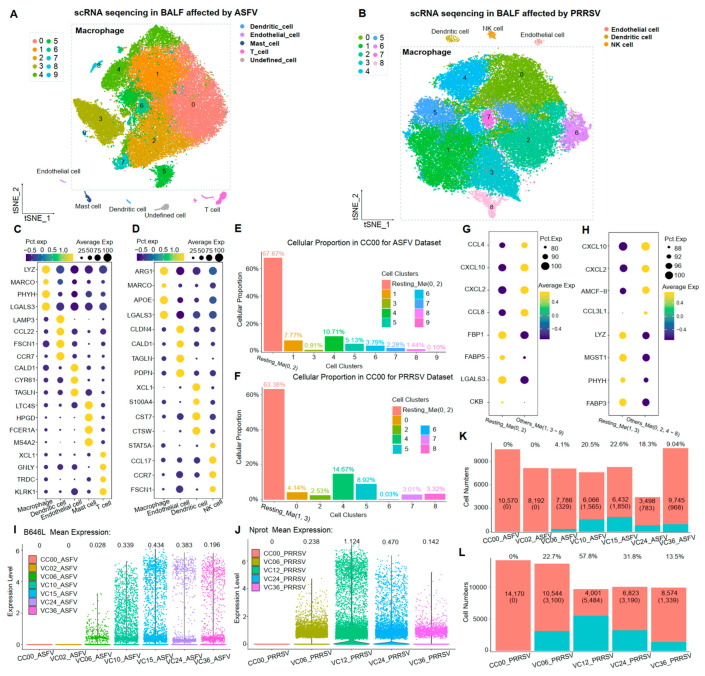
Single-cell transcriptome landscape in ASFV-infected and PRRSV-infected BALF Cells. (**A**,**B**) The TSNE plots of BALF cells in the ASFV-infected dataset and PRRSV-infected dataset, colored to show cluster information. (**C**,**D**) The bubble charts of the marker gene expression levels for identifying cell types in the ASFV-infected dataset and PRRSV-infected dataset, with brightness indicating log-normalized average expression and circle size indicating the percentage expressed. (**E**,**F**) Proportion of each cellular cluster in CC00 sample for two datasets. (**G**,**H**) The bubble charts of the top five gene expression levels in CC00 cellular clusters for two datasets. (**I**,**J**) Violin diagram of the expression levels for B646L and Nprot at different infection times. (**K**,**L**) The cell numbers and the proportions of B646L-expressing cells and of N-expressing cells at different infection times.

BALF cells were consistently collected from the same sacrificed healthy pig in each viral-infected experiment. Following sequencing and initial quality control, we obtained 12 single-cell transcriptomes comprising a total of 117,139 cells. These cells were categorized into different samples, including 10,872 cells from the CC00_ASFV sample (control sample without virus-infected in ASFV dataset), 8397 cells from the VC02_ASFV sample (ASFV-infected sample for 2 h), 8457 cells from the VC06_ASFV sample (ASFV-infected sample for 6 h), 7905 cells from the VC10_ASFV sample (ASFV-infected sample for 10 h), 8521 cells from the VC15_ASFV sample (ASFV-infected sample for 15 h), 4425 cells from the VC24_ASFV sample (ASFV-infected sample for 24 h), 11,025 cells from the VC36_ASFV sample (ASFV-infected sample for 36 h), 14,229 cells from the CC00_ PRRSV sample (control sample without virus-infected in PRRSV dataset), 13,711 cells from the VC06_ PRRSV sample (PRRSV-infected sample for 6 h), 9527 cells from the VC12_ PRRSV sample (PRRSV-infected sample for 12 h), 10,072 cells from the VC24_ PRRSV sample (PRRSV-infected sample for 24 h), and 9998 cells from the VC36_ PRRSV sample (PRRSV-infected sample for 36 h) (Table 1). To identify cell identities in BALF cells, dimensional reduction analyses with t-Distributed Stochastic Neighbor Embedding (t-SNE) were performed in each viral-infected dataset by analyzing the integrated cell-by-gene expression matrix. In the ASFV dataset, macrophages, dendritic cells, endothelial cells, mast cells, and T cells were identified in the BALF cells (Figure 1A), consistent with the previous report [24]. Different cellular clusters were grouped based on the expression of marker genes (Figure 1C). A similar result was observed in the PRRSV dataset (Figure 1B). The BALF cells in the PRRSV dataset consisted of macrophages, dendritic cells, endothelial cells, and NK cells, with several significantly differentially expressed genes for identifying these cell types (Figure 1D).

The percentage of porcine alveolar macrophages (PAMs) exceeded 96% of BALF cells in both datasets, with 57,694 cells in the ASFV dataset and 57,234 cells in the PRRSV dataset. Subsequently, cellular subpopulation clustering was performed again for these PAMs, both with the parameter resolution = 0.4, yielding 10 cell clusters (0–9) and nine cell clusters (0–8) in the ASFV and PRRSV datasets, respectively (Figure 1A,B). We observed that cell clusters 0 and 2 in the ASFV dataset and cell clusters 1 and 3 in the PRRSV dataset were predominantly present in uninfected control samples (CC00) (Figure 1E,F). The top 25 KEGG enrichment terms of the differential expression genes (DEGs) in cellular subclusters (0, 2) and cellular subclusters (1, 3) showed a higher correlation with the functions existing in M0-type macrophages, implying that these clusters were M0 PAMs (Figure S1A,B). Moreover, more than 30% of cells in CC00 still exhibited an inflammatory reaction, with chemokines of the CCL and CXCL families being significantly expressed in these cells (Figure 1G,H). This finding suggests that even virus-uninfected PAMs could exhibit an inflammatory state.

### 3.2. Expressions of Viral Capsid Proteins for ASFV and PRRSV in PAMs

The transcription of viral capsid proteins is a key indicator of the completion of viral replication within host cells. The p72 protein (gene: B646L) and the N protein (gene: Nprot) are primary viral capsid proteins in ASFV and PRRSV [6,7]. In this study, by investigating the expression of these two viral genes at different infection times in two datasets, we observed that in the ASFV dataset, the transcript of B646L was not detected in host cells at 2 h post-infection, only emerging for the first time at 6 h post-infection. Subsequently, expressions gradually increased, peaking at 15 h post-infection, followed by a subsequent decrease (Figure 1I). A similar pattern was observed in PRRSV infection, where the expression of Nprot was highest in the samples at 12 h of infection (Figure 1J). Interestingly, the proportion of cells expressing these two viral capsid proteins followed a similar tendency as the viral infection persisted. Notably, the proportion of B646L-expressing cells in ASFV infection was much lower than the proportion of N-expressing cells in PRRSV infection (Figure 1K,L).

In summary, we analyzed all infected and uninfected BALF cells in ASFV and PRRSV datasets, discerning various types of cells. Additionally, we characterized a host cell that was entirely inflamed, determining this status based on the expression of viral capsid proteins. We propose that the immune response of these host cells was exclusively triggered by viral infection. To dissect such immune responses, we utilized PAMs with no inflammatory response in CC00 as a negative control, combining them with the PAMs expressing the viral capsid proteins for downstream analyses (Figure S1C,D).

### 3.3. Common Immune Responses of PAMs Caused by the Infection of ASFV and PRRSV

**Figure 2:**
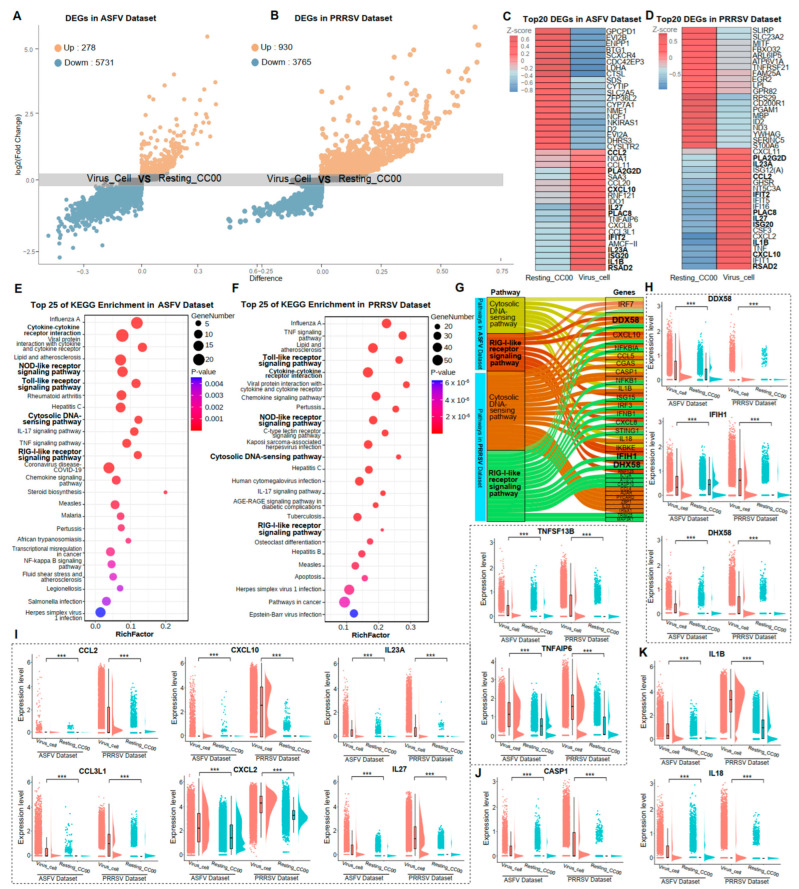
Common immune responses of PAMs caused by the infection of ASFV and PRRSV. (**A**,**B**) The volcanic map of single-cell transcriptome difference analysis for Virus_Cell vs. Resting_CC00 in the ASFV dataset and PRRSV dataset, respectively. Each point in the figure represents each gene. The X-axis represented the value of difference, which is the difference between pct.1 and pct.2, where pct.1 represented the average expression percentage of the gene in the cellular group of Virus_Cell, and pct.2 represented the average expression percentage of the gene in the cellular group of Resting_CC00. The closer the absolute value of difference was to 1, the greater the difference of the gene in the two subgroups. The Y-axis represented the log2 (Fold Change) for genes, with different colors showing different Log2FC, such as grey indicating genes with log2FC between −0.25 and 0.25, blue indicating genes with log2FC less than −0.25, and orange indicating genes with log2FC greater than 0.25. (**C**,**D**) Heatmap showing the top 20 genes’ expression levels of cellular groups Virus_Cell and Resting_CC00 in the ASFV dataset and PRRSV dataset, respectively, with brightness indicating the z-score of average expression and bolded fonts representing consistent genes in both datasets. (**E**,**F**) The top 25 KEGG terms with the most significant *p*-values among the cellular groups of Virus_Cell in two datasets, respectively, with each bubble in the y-axis representing each term and the x-axis representing these terms’ RichFactor, which was calculated by the ratio of the number of genes enriched in each term to the total number of genes in that term. The color intensity of each bubble indicated the *p*-values of the corresponding KEGG term, and the size corresponded to the gene numbers enriched in each term. (**G**) The up-regulated genes in the cellular groups of Virus_Cell in two datasets for the significant KEGG pathway “Cytosolic DNA-sensing pathway” and “RIG-I-like receptor signaling pathway,” respectively. (**H**–**K**) The ridge plots of DDX58, IFIH1, DHX58, CCL2, CCL3L1, CXCL10, CXCL2, IL23A, IL27, TNFSF13B, TNFAIP6, CASP1, IL-1B, and IL-18 in the cellular groups of Virus_Cell and Resting_CC00 in two datasets, respectively. Significant differences were denoted by *** (*p* < 0.001).

In this study, two distinct states of PAMs were defined: Virus_Cell, representing PAMs with completed viral replication, and Resting_CC00, representing PAMs with a complete absence of an inflammatory response. In the ASFV dataset, Virus_Cell comprised 5495 cells, and Resting_CC00 comprised 7174 cells. In the PRRSV dataset, Virus_Cell included 13,113 cells, and Resting_CC00 included 8981 cells (Figure S1C,D). The identification of DEGs induced by viral infections provided insights into the immune responses of host cells. Volcano plots of DEGs were shown in Figure 2A,B. We identified 278 and 930 significantly up-regulated genes in ASFV and PRRSV infections, respectively. Notably, the top 20 DEGs in the Virus_Cell group were identical for both datasets comprising 10 common genes, while none were shared in the Resting_CC00 group (Figure 2C,D), suggesting a consistent response of PAMs to these two distinct viruses. To further support this observation, KEGG enrichment analysis for these DEGs was performed. We detected 14 common pathways among the top 25 KEGG terms in both datasets, unveiling promising discoveries within these shared pathways (Figure 2E,F).

We described the common immune responses of PAMs during ASFV and PRRSV infections based on the four consistent pathways and the critical genes within them. The “Cytosolic DNA-sensing pathway” and “RIG-I-like receptor signaling pathway” were notably activated, both specifically recognizing viral nucleic acids and eliciting innate immune responses in host cells (Figure 2G). Three primary receptors for detecting viral nucleic acids, RIG-I (DDX58), MDA5 (IFIH1), and LGP2 (DHX58), were significantly higher expressed in the Virus_Cell group than in the Resting_CC00 group during ASFV and PRRSV infection (Figure 2H). As innate immune responses were activated, the “Toll-like receptor signaling pathway” and “NOD-like receptor signaling pathway” were also significantly activated, prompting host cells to produce multiple cytokines. We revealed that numerous cytokines from the CCL, CXCL, IL, and TNF families were up-regulated in expression among infected PAMs (Figure 2I). The “NOD-like receptor signaling pathway” could also trigger pyroptosis, as evidenced by the dramatic upregulation of CASP1, a marker gene for pyroptosis, in both ASFV and PRRSV infections, regulating the maturation of pro-inflammatory cytokines IL-1B and IL-18 (Figure 2J,K). These findings indicated that these four crucial pathways and pyroptosis constitute common immune responses in PAMs exposed to ASFV and PRRSV.

### 3.4. Gene Co-Expression Network of PAMs Exposed to Two Virus Infections

**Figure 3:**
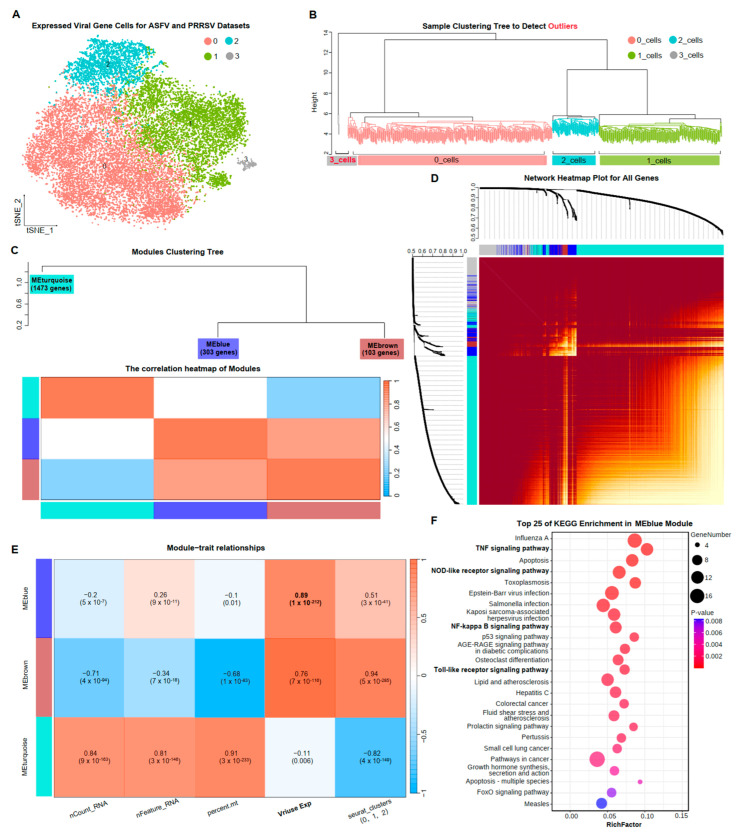
Gene co-expression network of PAMs exposed to two virus infections. (**A**) The TSNE plots of cells in the Virus_Cell group from ASFV and PRRSV datasets, colored to show cluster information. (**B**) The sample clustering tree to detect outliers, colored to show cluster information, with red indicating the outliers. (**C**) The modules’ clustering tree and the correlation heatmap of modules. (**D**) The network heatmap plot for all genes. (**E**) The correlation between cellular traits and gene modules by the Pearson correlation coefficient. (**F**) The top 25 KEGG terms with the most significant *p*-values among the 303 genes in MEblue, with each bubble in the y-axis representing each term and the x-axis representing these terms’ RichFactor, which was calculated by the ratio of the number of genes enriched in each term to the total number of genes in that term. The color intensity of each bubble indicated the *p*-values of the corresponding KEGG term, and the size corresponded to the gene numbers enriched in each term.

Co-expression of genes among cells reflected variations in cellular functions. To delve deeper into the common immune responses of PAMs under PRRSV and ASFV invasion, we performed an analysis of weighted gene co-expression network analysis (WGCNA) for all cells expressing viral capsid proteins in both datasets. We aggregated all 18,608 cells in the Virus_Cell group from ASFV and PRRSV datasets, resulting in a common count of 11,527 genes. Upon re-clustering based on this information, four (0–3) cellular subclusters emerged (Figure 3A). Sample clustering results indicated the need to exclude cellular subcluster 3 in downstream analyses (Figure 3B). Subsequently, we obtained three significant gene modules based on the pattern of intergenic expressions: module MEturquoise with 1473 genes, module MEblue with 303 genes, and module MEbrown with 103 genes (Figure 3C,D). Module MEblue and module MEbrown exhibited a strong correlation, while MEturquoise showed a weak correlation with both (Figure 3C).

Analyzing the correlations between modules and cellular traits, we revealed that module MEturquois, with the greatest number of genes, exhibited a high correlation of 0.91 with mitochondrial gene expression. This could suggest that genes in module MEturquois were associated with basal cellular functions. Additionally, a correlation of 0.94 was observed between module MEbrown and cellular subclusters. Notably, a 0.89 correlation was observed between module MEblue and viral capsid protein expression, implying some relevance between genes in module MEblue and viral replication (Figure 3E). The results of KEGG enrichment analyses for the 303 genes in module MEblue demonstrated that these co-expression genes were indeed involved in viral replication (Figure 3F). The “NOD-like receptor signaling pathway” and “Toll-like receptor signaling pathway” have been elucidated in detail in the results above, explaining their impact on viral infections. The WGCNA analysis among PAMs under ASFV and PRRSV infection further confirmed the existence of common immune responses in PAMs and identified 303 co-expressed genes related to these responses.

### 3.5. Negative Regulation of ASFV and PRRSV Genome Replication in PAMs

**Figure 4:**
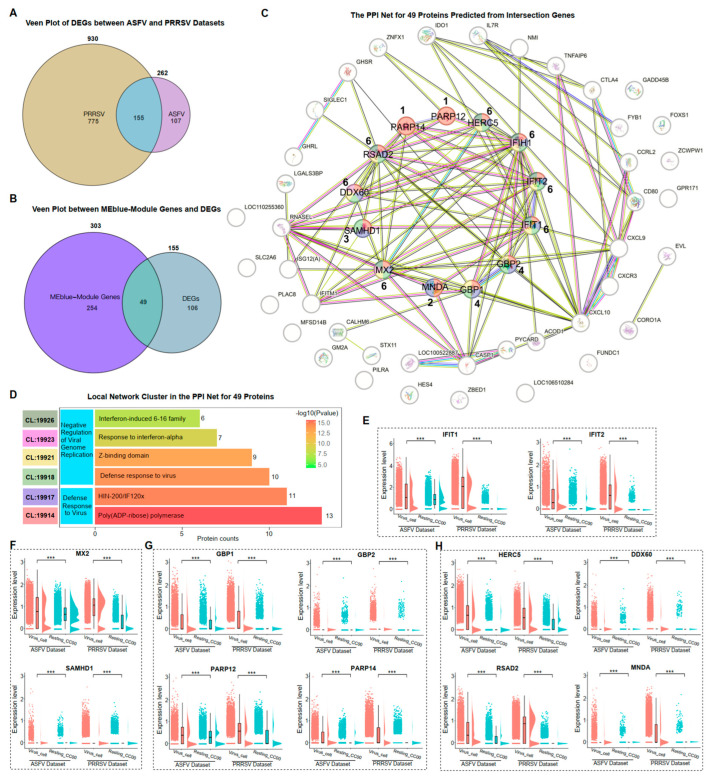
Negative regulation of ASFV and PRRSV genome replication in PAMs. (**A**) The veen plot of DEGs in the Virus_Cell group between ASFV and PRRSV datasets. (**B**) The veen plot between the MEblue-module genes and the common DEGs in both datasets. (**C**) The PPI network for 49 proteins predicted from the intersection genes from (**B**). (**D**) The local network cluster in the PPI network based on the biological processes (BP) of GO, colored to show the “CL” information and shown in (**C**) for those genes belonging with different “CL”. (**E**–**H**) The ridge plots of IFIH1, IFIT1, IFIT2, MX2, SAMHD1, GBP1, GBP2, PARP12, PARP14, HERC5, DDX60, RSAD2, and MNDA in the cellular groups of Virus_Cell and Resting_CC00 in two datasets, respectively. Significant differences were denoted by *** (*p* < 0.001).

Based on the expression of genes in single-cell transcriptomes, a co-expression module of genes significantly relevant to viral replication was identified. Since proteins are the direct-acting elements through which genes perform their functions in cells, our investigation aimed to determine whether the proteins translated by these co-expressed genes also had analogous interactions and functions in cells. 155 common DEGs were identified in PRRSV-infected and ASFV-infected PAMs (Figure 4A). There were 49 genes among the 303 co-expressed genes that were differentially expressed (Figure 4B). We then predicted the corresponding proteins according to these 49 genes and predicted the interactions between these proteins using STRING (Figure 4C). We found that 16 of these proteins had no interaction with the rest of the proteins. Clustering the remaining 33 interacting proteins based on the biological processes (BP) of Gene Ontology (GO), we revealed that two main biological processes were enriched: “Defense Response to Virus” and “Negative Regulation of Viral Genome Replication” (Figure 4D). Within the biological processes, we identified 13 interacting proteins, including IFIH1, IFIT1, IFIT2, MX2, SAMHD1, GBP1, GBP2, PARP12, PARP14, HERC5, DDX60, RSAD2, and MNDA (Figure 4C,D).

The expression of genes translating these 13 proteins in the ASFV and PRRSV datasets reflected the immune responses of virus-infected PAMs. IFIH1 has already been confirmed to be significantly higher expressed in the Virus_Cell group than in Resting_CC00 during ASFV and PRRSV infection (Figure 2H). Similar results were observed for the IFN-inducible genes IFIT1 and IFIT2 (Figure 4E). MX2 and SAMHD1 exhibited high expression levels. Moreover, the genes corresponding to the other 10 proteins with the function of inhibiting viral replication exhibited significantly higher expression in the Virus_Cell group than in the Resting_CC00 group among the two datasets (Figure 4F–H). In summary, we identified 13 proteins with the ability to inhibit viral replication through PPI analysis and confirmed the expression of corresponding genes.

### 3.6. Validation of Crucial Gene Expressions on PAMs by Two Viral Infections

**Figure 5:**
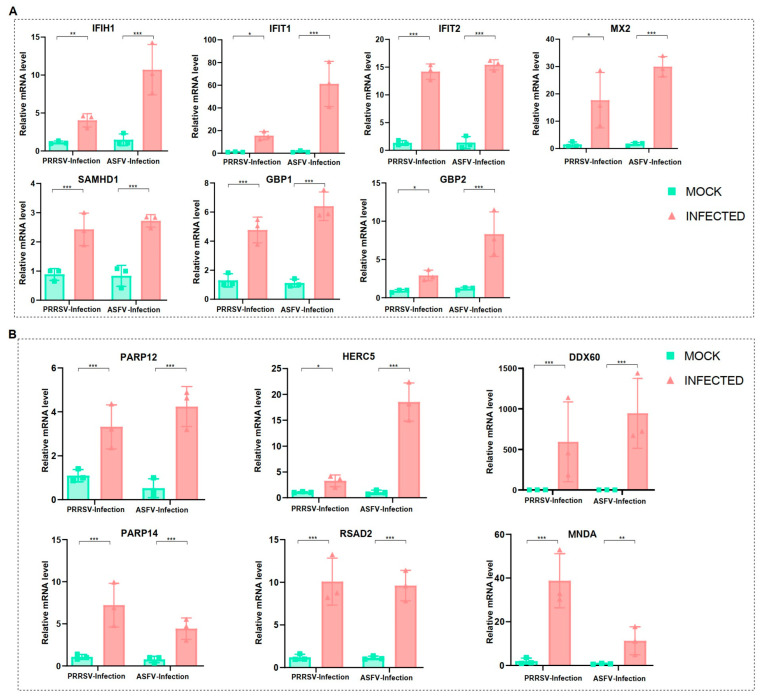
Validation of crucial gene expressions on PAMs by two viral infections. (**A**) The gene expressions of PAMs under PRRSV and ASFV infections from qRT-PCR results, including IFIH1, IFIT1, IFIT2, MX2, GBP1, GBP2, and SAMHD1. (**B**) The gene expressions of PAMs under PRRSV and ASFV infections from qRT-PCR results, including PARP12, PARP14, HERC5, DDX60, RSAD2, and MNDA. Significant differences were denoted by * (*p* < 0.05), ** (*p* < 0.01), and *** (*p* < 0.001).

To validate the above findings, we conducted in vitro infections of PAMs with PRRSV and ASFV (MOI = 1), utilizing the JAX1 strain for PRRSV and the HLJ/18 strain for ASFV. Subsequently, the transcriptional levels of 13 genes were measured through qRT-PCR. The results demonstrated a significant increase in the expression of these genes in the infected group compared to the mock group (Figure 5A,B). The consistent outcomes from both qRT-PCR and scRNA-Seq underscore the reliability of the two datasets employed in this study. In conclusion, we have confirmed that these 13 proteins (IFIH1, IFIT1, IFIT2, MX2, SAMHD1, GBP1, GBP2, PARP12, PARP14, HERC5, DDX60, RSAD2, and MNDA) were indeed overexpressed at the mRNA level as a consequence of both viral infections.

## 4. Discussion

In the present study, two single-cell transcriptome datasets of virus-infected BALF cells (one downloaded from a published publication and the other obtained from the experimental assay conducted in this study) comprehensively elucidated the common immune responses of PAMs infected with ASFV and PRRSV. We identified 13 proteins, translated by the host cells, with the ability to inhibit the viral replication. Less than 4% of the BALF cells in the two datasets were characterized as dendritic cells, endothelial cells, mast cells, NK cells, and T cells. These findings align with similar results obtained by extracting BALF from viral infection experiments *in vivo* and analyzing its cellular composition [30,31,32]. Macrophages can manifest diverse functional phenotypes in health and disease conditions, such as M0-type, pro-inflammatory, and anti-inflammatory subpopulations [33,34]. Here, the percentage of M0-type PAMs identified was highest in the uninfected samples in the two datasets. About 30% of the uninfected PAMs remained non-M0-type, perhaps because macrophages, serving as immune cells, would maintain an inflammatory state in response to an abnormal microenvironment in the organism.

Virus infections involve multi-step processes comprising entry, replication, assembly, and release of progeny. The signature of completed viral assembly is the translation of capsid protein in the host cell [35]. We classified PAMs into two groups, Virus_Cell and Resting_CC00, by utilizing the expression of viral capsid proteins and non-inflammatory cells in the CC00 samples. We revealed lots of consistency in genes and cellular functions in PAMs under ASFV and PRRSV infections. The RIG-I-like receptor family, composed of three homologous proteins, RIG-I, MDA5, and LGP2, belongs to pattern recognition receptors (PRRs) and can recognize foreign RNA, resulting in the production of antiviral cytokines and the establishment of a broadly effective cellular antiviral state [36]. Higher expressions of RIG-I (DDX58), MDA5 (IFIH1), and LGP2 (DHX58) in the Virus_Cell group were observed compared to the Resting_CC00 group during ASFV and PRRSV infection, suggesting that the PAMs have already recognized the exogenous virus. Meanwhile, the pathway “Cytosolic DNA-sensing pathway”, reported to recognize various RNA and DNA viruses for host cells, was significantly enriched in this study [37]. Once changes in the cellular microenvironment in PAMs under ASFV and PRRSV infection occurred, the various cytokine expressions would upregulate, such as CCL4, TNF-α, and CXCL10 [38,39,40]. We revealed that the CCL, CXCL, IL, and TNF families of cytokines were all up-regulated in expression among infected PAMs. Pyroptosis is a recently discovered form of inflammation-related programmed cell death, characterized by the activation of caspase-1 [41,42]. We discovered that CASP1, IL-1B, and IL-18a were dramatically up-regulated in both ASFV and PRRSV infections, which suggested the occurrence of pyroptosis. The above results were compatible with the findings of two existing publications on ASFV and PRRSV infections [43,44].

Investigators have shown that genes with similar functions frequently have similar patterns of mRNA expression [45,46]. We identified 303 co-expressed genes related to the common immune responses described above among PAMs under ASFV and PRRSV infection through the analysis of WGCNA. Furthermore, we identified 13 proteins predicted to inhibit viral genome replication based on their predicted interactions with the proteins translated from these genes. Seven of these proteins have been reported to inhibit ASFV and PRRSV replication; for example, IFIH1 could result in a series of immune responses by activating pattern recognition receptors (PRRs), and IFIT1, IFIT2, MX2, GBP1, GBP2, and SAMHD1 could inhibit viral replication by inducing the production of IFN in the host cells [47,48,49,50,51,52,53,54]. The other six proteins were reported to inhibit viral replication in other viral infections. PARP12 and PARP14, as interferon-induced genes (ISG), were found to block RNA translation of viruses and played a potential role in cellular defenses against viral infections [55,56]. In IAV infections, HERC5 and DDX60 could upregulate IFN-β and inhibit viral replication [57,58]. RSAD2 and MNDA were required for IFN-α induction in host cells to inhibit the virus [59,60]. ISG, IFN-α, and IFN-β were types of interferons (INFs) [61]. The effects of IFNs were exerted by binding to specific universally expressed cell-surface receptors, which activated the corresponding signaling pathways that inhibited viral replication, including the four common pathways: “Cytosolic DNA-sensing pathway”, “RIG-I-like receptor signaling pathway”, “Toll-like receptor signaling pathway”, and “NOD-like receptor signaling pathway” identified in this study [36,62,63,64]. Finally, we verified that the genes translating these proteins were indeed differentially expressed in the ASFV-infected and PRRSV-infected PAMs by qRT-PCR. However, the protein-level expression differences of these genes have not been validated in this work. In summary, we discovered 13 inhibitions of viral replication in PAMs under ASFV and PRRSV infections. However, the detailed mechanisms of these six proteins (PARP12, PARP14, HERC5, DDX60, RSAD2, and MNDA) for inhibiting viral replication in PAMs remains to be further investigated.

## 5. Conclusions

We have provided a comprehensive understanding of the common immune response of PAMs caused by the infection of ASFV and PRRSV at single-cell levels. Three PRRs (DDX58, IFIH1, and DHX58) particularly recognize ASFV and PRRSV in host cells and induced the up-regulated expression of four cytokines families (CCL, CXCL, IL, and TNF) and the development of pyroptosis. We identified thirteen proteins with the ability to inhibit viral replication, six of which were first discovered in PAMs for inhibiting the replication of ASFV and PRRSV.

## Figures and Tables

**Table 1:** The gene and cell numbers of ASFV and PRRSV datasets.

Dataset Name	Sample Name	Before Quality Control		After Quality Control	
		Gene Numbers	Cell Numbers	Gene Numbers	Cell Numbers
ASFV	CC00	13,620	12,761	13,620	10,872
	VC02	13,764	9266	13,764	8397
	VC06	13,997	10,309	13,997	8457
	VC10	14,128	9753	14,128	7905
	VC15	13,932	11,152	13,932	8521
	VC24	13,604	5267	13,604	4425
	VC36	13,764	12,347	13,764	11,025
PRRSV	CC00	13,121	14,832	13,121	14,229
	VC06	12,946	15,019	12,946	13,711
	VC12	12,477	10,990	12,477	9527
	VC24	12,127	10,331	12,127	10,072
	VC36	12,120	10,164	12,120	9998

**Table 2:** Sequences of qRT-PCR primers.

Gene	Transcript ID	Primer (5′-3′)	Title 3
RSAD2	ENSSSCG00000008648	F: CGAGTCTAACCGGCAGATGA	82 bp
		R: CTTCCGCCCGTTTCTACAGT	
HERC5	ENSSSCG00000030548	F: GGTTTCCTGCCAGGCCTAAA	148 bp
		R: TCCAACGATGGCTCTTGGTC	
PARP12	ENSSSCG00000016502	F: TCGGTCTTGGCCGCGT	150 bp
		R: ACTCCTACACTCCTTCCCGT	
PARP14	ENSSSCG00000011874	F: GCTGGCAAGAATGCGACTT	150 bp
		R: TCCAAGCGTGTAGGTTCCAG	
IFIH1	ENSSSCG00000015897	F: AGCCACAGATCAGCCAAGTC	208 bp
		R: AGCCACAGTCTCTTCATCTGAATC	
IFIT1	ENSSSCG00000010452	F: TGACTCACAGCAACCATGAGTAATA	146 bp
		R: TCAATCTCCTCCAAGACCCTG	
IFIT2	ENSSSCG00000010451	F: GCACAGCAATCATGAGTGAGAC	92 bp
		R: TCCCTCTACCAAGTTCCAGGT	
SAMHD1	ENSSSCG00000027806	F: TTTGCTGCACGACAGCATTTT	74 bp
		R: ACAACATCACCATCCTGTGGC	
MX2	ENSSSCG00000012076	F: GGGAAATACGCAAAGCCCAGG	110 bp
		R: GATGAGGGTCAGGTCTGGAAC	
DDX60	ENSSSCG00000009720	F: TCACAGACCCCGTGCTCAT	95 bp
		R: AGAAGACCCAGACATCTCTTCAT	
MNDA	ENSSSCG00000037532	F: TCCAGGTGGGAACTCGATAG	128 bp
		R: ACATTGTGCTCTCTTCCCACC	
GBP1	ENSSSCG00000024973	F: GGGGGATGTTGAGAAGGGTG	768 bp
		R: TGCTCAGGAGAATTGCCAGG	
GBP2	ENSSSCG00000006923	F: AAACTGCAAAGATCCTGTCTGC	128 bp
		R: GCCCAGAGAGAAGCCCTTGTT	
GAPDH	ENSSSCG00000000694	F: TGACAACAGCCTCAAGATCG	51 bp
		R: GTCTTCTGGGTGGCAGTGA	

## Data Availability

The data presented in this study are available on request from the corresponding author due to the privacy.

## Supplementary Materials

The following supporting information can be downloaded at: https://www.mdpi.com/article/10.3390/microorganisms12030563/s1, Figure S1: KEGG enrichment analysis and cell grouping in ASFV and PRRSV datasets.
