# Supplementary material for: Unveiling Shared Immune Responses in Porcine Alveolar Macrophages during ASFV and PRRSV Infection Using Single-Cell RNA-seq
Source: Microorganisms. 2024 Mar 12;12(3):563. doi: 10.3390/microorganisms12030563 (PMC10974629; doi:10.3390/microorganisms12030563)
Supplement: Supplementary file 1 [file microorganisms-12-00563-s001.zip › microorganisms-2860225-supplementary.pdf]

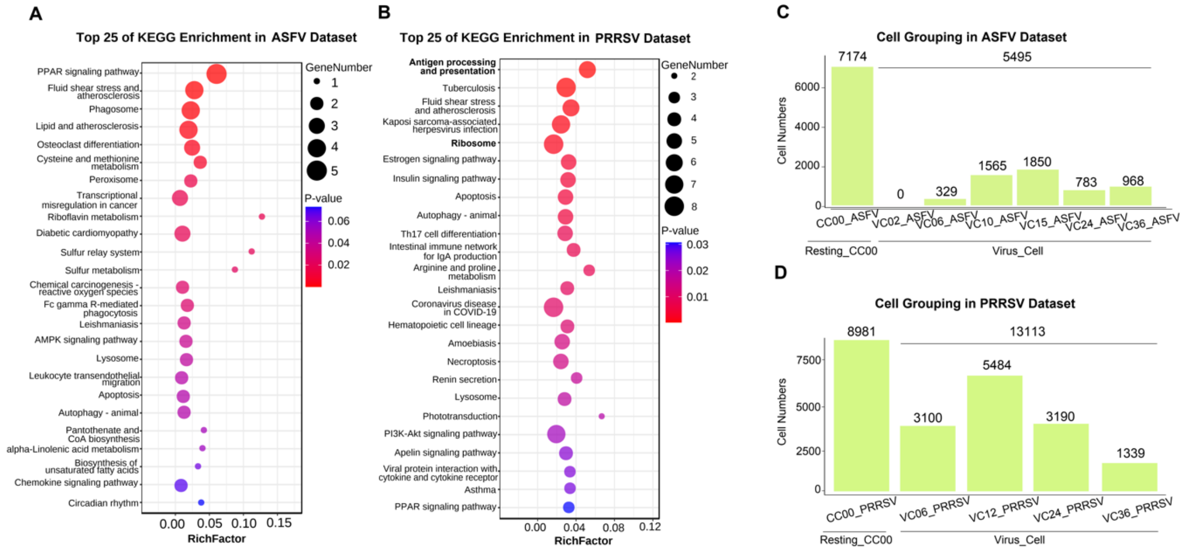

Figure S1. KEGG Enrichment Analysis and Cell grouping in ASFV and PRRSV Datasets. (A, B) The top 25 KEGG terms with the most significant P-values among the cellular groups of cellular subclusters (0, 2) and cellular subclusters (1, 3) in two datasets respectively, with each bubble in the y-axis representing each term and the x-axis representing these terms' RichFactor, which was calculated by the ratio of the number of genes enriched in each term to the total number of genes in that term. The color intensity of each bubble indicated the P-values of the corresponding KEGG term, and the size corresponded to the gene numbers enriched in each term. (C, D) The cellular grouping results by expressing two viral capsid proteins in two datasets respectively.
